# Editorial: Principles and Clinical Applications of Interstitial Brachytherapy

**DOI:** 10.3389/fonc.2022.877165

**Published:** 2022-03-09

**Authors:** Alexis Vrachimis

**Affiliations:** ^1^ Department of Nuclear Medicine, German Oncology Center, University Hospital of the European University, Limassol, Cyprus; ^2^ Cancer Research and Innovation Center (CARIC), Limassol, Cyprus

**Keywords:** brachytherapy (BT), high dose rate (HDR), low dose rate (LDR), EBRT, PSMA

Brachytherapy (BT) is a powerful minimally invasive therapeutic modality of contemporary radiation oncology that exhibits several advantages in experienced hands, such as high-precision, hypofractionation and dose-escalation. In this Frontiers Research Topic, further insight is provided into the principles and clinical applications of interstitial brachytherapy also with the implementation of novel imaging agents (Tsechelidis and Vrachimis).

An introductory article from Roussakis and Anagnostopoulos present the physical and dosimetric aspects of the iridium (^192^Ir) application in high-dose-rate (HDR) BT for different anatomical sites as well as the corresponding dosimetric comparison with the stereotactic body radiation therapy (SBRT) techniques.

In the same wavelength, Wei et al. discuss this approach using sealed sources/radioactive seeds (namely ^125^I) for interstitial “permanent” (half-life of 60 days: low-dose rate, LDR) BT and summarize the recent advances of ^125^I BT in cancer therapy, which cover experimental research to clinical investigations, including the development of novel techniques. Namely, they emphasize on the fact that with BT the extremely high doses achieved are by far higher than external beam radiotherapy (EBRT), with the added benefit that doses drop off rapidly outside the target lesion by minimizing the exposure of uninvolved surrounding normal tissue. Furthermore, over the past few decades the seed implantation evolved from ultrasound guidance to computed tomography guidance. Additionally, recent technical innovations such as 3D-printed individual templates, BT treatment planning systems, and artificial intelligence navigator systems remarkably increased the accuracy of “tailored-made” brachytherapy also by utilizing ^125^I to deliver high doses in carcinomas of hollow cavity organs by the utility of self-expandable metal stents.

Walter et al. provide a single-center experience from Germany in HDR BT and SBRT as bridging therapy (even up to more than 2 years) to liver transplantation (LT) in 14 hepatocellular carcinoma (HCC) patients with very promising results. Even more interesting was the fact that eight patients were in complete remission after histological examination of the explanted liver, while only four liver specimens showed vital tumor. Additionally, no hepatic HCC recurrence occurred following LT in a median follow-up of more than 1 year. Concluding the authors state that SBRT and BT can close the gap for patients not suitable for other locally ablative treatment options and are feasible and well tolerated as bridging to LT.

Gynecological tumors are a further area that brachytherapy plays a pivotal role. Itami et al. present a well-structured overview of combined interstitial and intracavitary HDR BT of cervical cancer. Emphasis is given to this combination of BTs (so called “hybrid” BT) that can increase the dose to large parametrial involvements without increasing the dose to the rectum and bladder. The authors present an overview from a phase I/II study of hybrid brachytherapy that was launched in Japan in 2015. Since then, more than 120 patients received this “hybrid” regime in Japan safely and with a high quality of radiation.

A very interesting technical note from Japan is presented from Shimizu et al. in collective of nine patients with cervical or endometrial cancer receiving HDR BT. As stated in the paper, this group uses real-time transrectal ultrasonography (TRUS) to guide freehand interstitial needle insertion. However, this technique is hampered for target tumors located deeper beyond the rectum that are visible to CT. Thus, the authors describe their experience in combining TRUS and transabdominal ultrasonography (TR/TA-US). In a median follow-up period of 15 months, only two infield recurrences were registered, thus proving the feasibility of the novel technique introduced for deeply situated gynecologic malignancies (out of reach of TRUS).

Overall, the Research Topic provides an overview of available data around BT. While enthusiasm for its use exists, much work remains to be done especially when it comes to its comparison with EBRT, as the availability of BT is spared for the experienced hands. Hopefully, the topic will inspire more investigators to explore this area and encourage its implementation.

## Author Contributions

The author confirms being the sole contributor of this work and has approved it for publication.

## Conflict of Interest

The author declares that the research was conducted in the absence of any commercial or financial relationships that could be construed as a potential conflict of interest.

## Publisher’s Note

All claims expressed in this article are solely those of the authors and do not necessarily represent those of their affiliated organizations, or those of the publisher, the editors and the reviewers. Any product that may be evaluated in this article, or claim that may be made by its manufacturer, is not guaranteed or endorsed by the publisher.

